# Autonomous multi-joint soft exosuit with augmentation-power-based control parameter tuning reduces energy cost of loaded walking

**DOI:** 10.1186/s12984-018-0410-y

**Published:** 2018-07-13

**Authors:** Sangjun Lee, Jinsoo Kim, Lauren Baker, Andrew Long, Nikos Karavas, Nicolas Menard, Ignacio Galiana, Conor J. Walsh

**Affiliations:** 1 John A. Paulson School of Engineering and Applied Sciences, Harvard University, Cambridge, MA USA; 2 Wyss Institute for Biologically Inspired Engineering, Harvard University, Cambridge, MA USA

**Keywords:** Exosuit, Assistance, Control, Tuning, Augmentation power, Metabolic cost

## Abstract

**Background:**

Soft exosuits are a recent approach for assisting human locomotion, which apply assistive torques to the wearer through functional apparel. Over the past few years, there has been growing recognition of the importance of control individualization for such gait assistive devices to maximize benefit to the wearer. In this paper, we present an updated version of autonomous multi-joint soft exosuit, including an online parameter tuning method that customizes control parameters for each individual based on positive ankle augmentation power.

**Methods:**

The soft exosuit is designed to assist with plantarflexion, hip flexion, and hip extension while walking. A mobile actuation system is mounted on a military rucksack, and forces generated by the actuation system are transmitted via Bowden cables to the exosuit. The controller performs an iterative force-based position control of the Bowden cables on a step-by-step basis, delivering multi-articular (plantarflexion and hip flexion) assistance during push-off and hip extension assistance in early stance. To individualize the multi-articular assistance, an online parameter tuning method was developed that customizes two control parameters to maximize the positive augmentation power delivered to the ankle. To investigate the metabolic efficacy of the exosuit with wearer-specific parameters, human subject testing was conducted involving walking on a treadmill at 1.50 m s^− 1^ carrying a 6.8-kg loaded rucksack. Seven participants underwent the tuning process, and the metabolic cost of loaded walking was measured with and without wearing the exosuit using the individualized control parameters.

**Results:**

The online parameter tuning method was capable of customizing the control parameters, creating a positive ankle augmentation power map for each individual. The subject-specific control parameters and resultant assistance profile shapes varied across the study participants. The exosuit with the wearer-specific parameters significantly reduced the metabolic cost of load carriage by 14.88 ± 1.09% (*P* = 5 × 10^− 5^) compared to walking without wearing the device and by 22.03 ± 2.23% (*P* = 2 × 10^− 5^) compared to walking with the device unpowered.

**Conclusion:**

The autonomous multi-joint soft exosuit with subject-specific control parameters tuned based on positive ankle augmentation power demonstrated the ability to improve human walking economy. Future studies will further investigate the effect of the augmentation-power-based control parameter tuning on wearer biomechanics and energetics.

**Electronic supplementary material:**

The online version of this article (10.1186/s12984-018-0410-y) contains supplementary material, which is available to authorized users.

## Introduction

Lower-limb assistive devices have been designed to assist with human locomotion [1–12]. Recently, different groups have used rigid but lightweight mechanisms to create low-profile exoskeletons assisting with a specific target joint, and studies have shown that these devices may substantially reduce the energy cost of loaded [6] and unloaded [7–12] walking. For example, Lee et al. showed that their hip exoskeleton reduced the metabolic cost of walking by 21% compared to walking without wearing the device [12]. For ankle, Mooney et al. reported an 11% net benefit for walking [10] and an 8% net benefit for load carriage [6] using their autonomous ankle exoskeleton.

Our group has been developing soft exosuits that use functional textiles to anchor to the body and deliver assistance in parallel with the underlying muscles [13–20]. In studies with tethered versions of the device, exosuits have been shown to significantly reduce the energy cost of regular walking [17, 20], walking with load [16, 19], and running [18]. For an autonomous version, Panizzolo et al. showed a 7% net metabolic reduction for loaded walking compared to equivalent-mass-removed condition (walking with the device unpowered but removing the equivalent mass of the device) [15].

Over the past few years, there has been a growing recognition on the importance of control individualization for such gait assistive devices to maximize one’s benefit; however, only a few studies so far have investigated methods to systematically customize the controller of assistive devices. Conventionally, researchers have used manual tuning to individualize the assistance of exoskeletons [12] or powered prostheses [3], where the wearer or an external operator subjectively tunes the control parameters based on the user’s perception or the observation of gait kinematics/kinetics. A challenge with a manual parameter tuning process is that it can involve a significant level of human subjective intervention, thus requiring expert knowledge and experience with the hardware. A more recent approach is human-in-the-loop optimization, where an optimization algorithm finds the optimal parameters that maximize one’s metabolic benefit, estimating the wearer’s instantaneous metabolic cost while walking [11, 20–22]. This approach holds advantages in that it automatically optimizes control parameters by directly monitoring the user’s metabolic cost; however, the current approach requires a user to wear respiratory measurement equipment throughout the process. The field of prosthetics has made efforts to bridge the gap between these two approaches [23–27]. Researchers have derived dynamic models of locomotion with specific types of powered prostheses and used computational algorithms, such as supervised learning [24], extremum seeking controller (ESC) [25], or adaptive dynamic programming (ADP) [26], to find optimal impedance control parameters in the model for each individual. Among them, Huang et al. suggested a method called cyber-expert tuning system for a powered knee prosthesis, where they implemented several decision rules of manual tuning into a computational algorithm based on data from the device’s own wearable sensors [27]. The approach of performing automatic parameter tuning with only device sensors is appealing as it opens the door to this being performed outside of a lab setting. However, it remains unclear how this approach can be applied to the devices augmenting the gait of healthy individuals, because it is currently unclear what may be proxy objective metrics for metabolic cost and how those metrics can be measured by body-worn sensors. Therefore, if a control tuning method can be developed based on an objective function that is easily measurable and strongly correlated with metabolic cost, it may greatly improve the energetic efficacy of a gait assistive device for healthy individuals.

In this paper, we present an updated version of the autonomous multi-joint soft exosuit aimed at overground walking in outdoor settings [28]. In addition, we propose an online parameter tuning method that automatically customizes assistance based on the positive power delivered to the ankle by the exosuit. This is based on the assumption that a positive correlation exists between the positive ankle augmentation power and the corresponding metabolic benefit [6, 10, 19, 29–33]. Given that this proxy objective metric can easily be measured by wearable sensors, we believe this augmentation-power-based parameter tuning approach holds a promise, given the desire to enable control individualization in unconstrained environments. Additionally, we present results from human subject testing demonstrating the metabolic efficacy of the soft exosuit with the subject-specific control parameters during loaded walking.

## Methods

### Soft exosuit

**Fig. 1:**
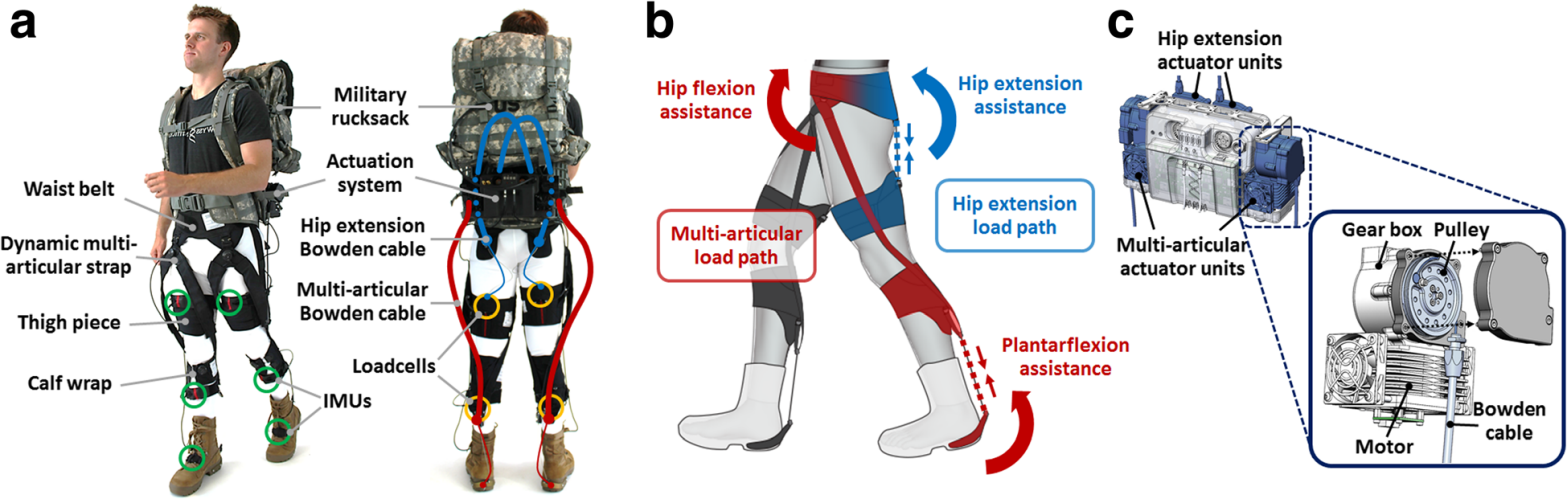
The autonomous multi-joint soft exosuit used in this study. **a** The apparel components and the hardware implementation. Bowden cable routings for multi-articular assistance and hip extension assistance are indicated by red and blue lines, respectively (thick lines: cable sheaths, thin lines: inner cables). Sensor placements for IMUs and loadcells are marked by green and yellow circles, respectively. **b** Two load paths specified by the textile architecture: multi-articular load path assisting with plantarflexion and hip flexion (highlighted in red) and hip extension load path assisting with hip extension (highlighted in blue). **c** A 3-D CAD model of the mobile actuation system consisting of four independent actuator units (highlighted in blue) and an exploded view of a multi-articular actuator unit

The multi-joint soft exosuit is designed to assist with plantarflexion, hip flexion, and hip extension while walking [28]. As shown in Fig. 1a, the exosuit apparel components consist of a waist belt, two thigh pieces, two calf wraps, two dynamic multi-articular straps connecting the front of the waist and the back of the calf, and two boot covers wrapping around the wearer’s ankle. The multi-articular strap is designed to distribute assistive forces applied at the ankle between the calf wrap and the waist belt [28]. The total mass of all suit components for a size medium is 1.1 kg, including two metal brackets bolted to the back of military boots. As shown in Fig. 1b, this textile architecture creates two different load paths on each leg: a multi-articular load path assisting with plantarflexion and hip flexion during push-off and a hip extension load path assisting with hip extension in early stance.

### Hardware implementation

A mobile actuation system was developed to generate assistive forces and mounted at the lower back of a military rucksack [28]. As shown in Fig. 1c, the actuation system consists of four independent actuator units, two for multi-articular load path and the other two for hip extension load path. Each actuator unit is comprised of an Emoteq frameless 6-pole motor (Allied Motion Inc., Amherst, NY, USA), a Spiroid helicon gear box (38:1 gearing ratio for multi-articular actuator units and 36:1 for hip extension actuator units; Illinois Tool Works, Inc., Glenview), and a 55-mm diameter multi-wrap pulley. The forces generated by the actuation system are transmitted to the exosuit via Bowden cables; when the motor retracts the Bowden cable, the distance between two attachment points on the exosuit is shortened, creating assistive forces along the corresponding load path. The actuation system including Bowden cables weighs 5.9 kg, and a 48 V-8 A∙hr. Li-Po battery pack (2.0 kg) stowed in the rucksack was used to power the actuation system that would be sufficient for approximately 8 km of continuous walking operation.

On each leg, a linear daisy-chain harness including three inertial measurement units (IMU; MTi-3 AHRS; Xsens Technologies B.V., Enschede, Netherlands) and two load cells (LSB200; Futek Advanced Sensor Technology Inc., Irvine, CA, USA) was placed to collect real-time data from the exosuit and the wearer. As shown in Fig. 1a, the IMUs were attached to the wearer’s thigh, shank, and foot to measure the sagittal-plane orientation and the angular velocity of each segment, while the load cells were mounted in series with the Bowden cables to monitor the level of assistive force delivered to the wearer through the exosuit. The full sensor harnesses including all sensors weigh 0.3 kg.

### Biologically inspired control

**Fig. 2:**
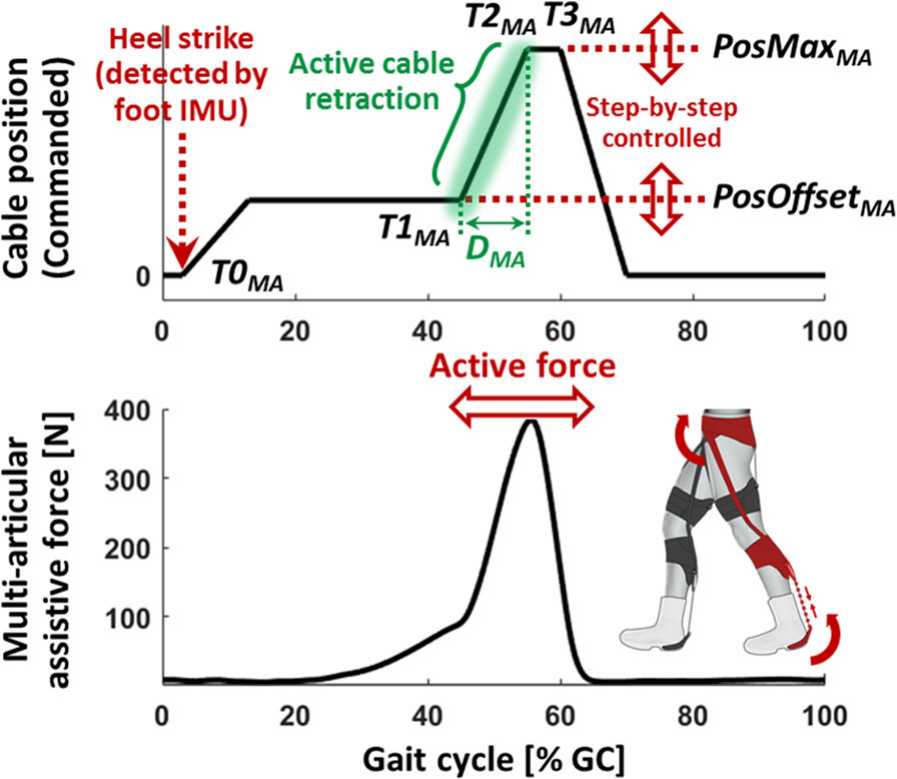
Representative data for the multi-articular controller: a Bowden cable position profile (top) and a resultant assistive force profile (bottom). The multi-articular assistance starts from the heel strike detected by the foot IMU, and delivers the majority of assistance during push off. The active cable retraction phase (highlighted in green) was parameterized into T1_MA_ and D_MA_ (T2_MA_ - T1_MA_), and these parameters were customized by the augmentation-power-based control parameter tuning method

**Fig. 3:**
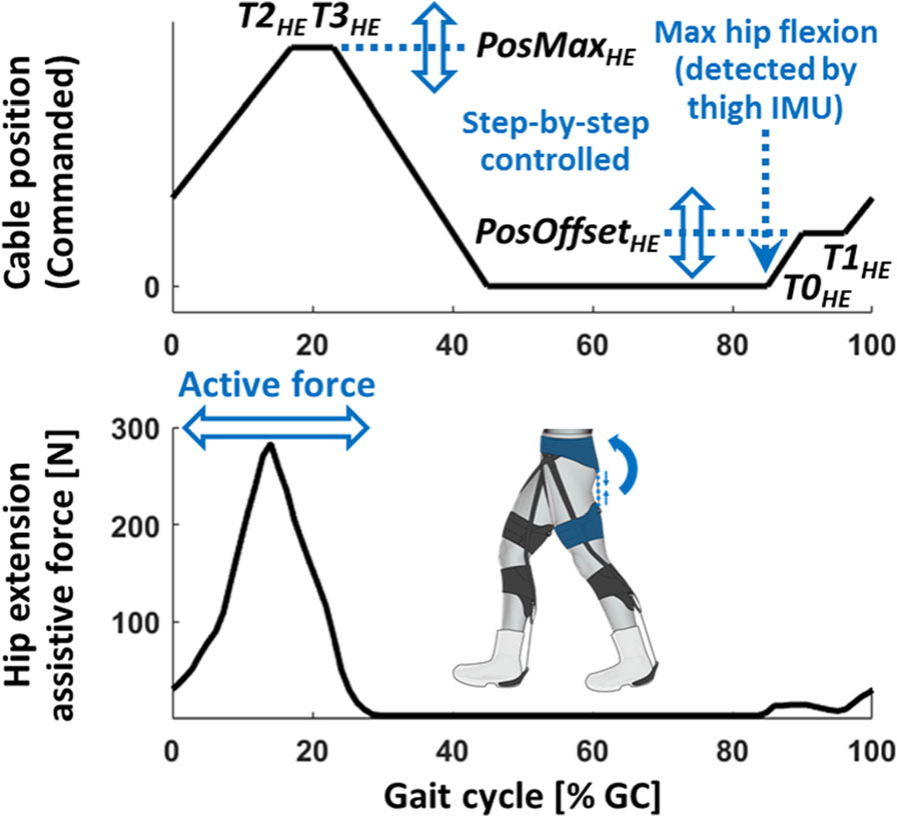
Representative data for the hip extension controller: a Bowden cable position profile (top) and a resultant assistive force profile (bottom). The hip extension assistance starts from the maximum hip flexion detected by the thigh IMU, and delivers the active assistance in early stance

As with the previous-version exosuits [34, 35], the controllers for the multi-articular and hip extension load paths both performed a force-based position control of the Bowden cables to generate assistive forces. Inspired by biological behavior of the target joints, the controller applies the assistance by retracting the Bowden cable during a target period within a walking cycle. As shown in Figs. 2 and 3, the cable position profiles were defined by four timing parameters, ***T0***, ***T1***, ***T2***, and ***T3***, which are represented as percentage of a gait cycle (% GC) and two cable position parameters, ***PosOffset*** and ***PosMax***, which are iteratively adjusted on a step-by-step basis [34, 35]:

- ***T0***: the start timing of the controller within a gait cycle.
- ***T1***: the onset timing of the active cable retraction.
- ***T2***: the completion timing of the active cable retraction.
- ***T3***: the start timing of the cable release.
- ***PosOffset***: Bowden cable position right before the active cable retraction (at ***T1***).
- ***PosMax***: Bowden cable position when the cable is fully retracted (from ***T2*** to ***T3***).

#### Multi-articular controller (MA)

The controller for the multi-articular load path was designed to deliver the majority of assistance during ankle push-off [34]. As shown in Fig. 2, the controller first detected the heel strike using the first peak of sagittal-plane gyro signal from the foot IMU, which happens approximately at 5% GC [34]. This event was used as the start of the multi-articular controller, ***T0***_***MA***_ (i.e. ***T0***_***MA***_ = 5% GC), and starting from ***T0***_***MA***_, the motor shortened the cable up to ***PosOffset***_***MA***_ at a constant speed of 394 mm s^− 1^ (65% of the maximum cable speed of the multi-articular actuator unit). Note that this cable speed was determined during pilot experiments to strike balance between being fast enough to reach ***PosOffset***_***MA***_ during early stance and not being excessively fast to not restrict the wearer’s dorsiflexion. After reaching ***PosOffset***_***MA***_, the controller maintained this cable position until the onset of the active cable retraction, ***T1***_***MA***_ (automatically tuned; details in the following section). Then, the cable was actively retracted up to ***PosMax***_***MA***_ until the completion timing of the active cable retraction, ***T2***_***MA***_ (automatically tuned; details in the following section). Then, the controller held the cable position constant until the load cell detects a force drop as the ankle further plantarflexes, and this event was used as the start of the cable release, ***T3***_***MA***_. Starting from ***T3***_***MA***_, the motor released the cable at the maximum speed of 606 mm s^− 1^ to its zero position where the cable is completely slack, in order to not restrict the wearer during swing phase. After reaching the zero position, the motor maintained this cable position until the next heel strike detection.

At the end of each stride, the controller either increased or decreased ***PosOffset***_***MA***_ and ***PosMax***_***MA***_ for the next stride by comparing the desired and the measured force. For example, ***PosOffset***_***MA***_ was adjusted to deliver a peak force of 75 N (equivalent joint moment of approximately 7.5 Nm) between ***T0***_***MA***_ and ***T1***_***MA***_, to consistently pretension the cable before the active cable retraction. ***PosMax***_***MA***_ was adjusted to deliver a peak force of 400 N (equivalent joint moment of approximately 40 Nm) between ***T1***_***MA***_ and ***T3***_***MA***_, as a primary means to deliver assistance during the active cable retraction.

#### Hip extension controller (HE)

The controller for the hip extension load path aimed at applying assistance in early stance while hip extensor muscles are active. The hip extension controller used constant timing parameters (***T0***_***HE***_, ***T1***_***HE***_, ***T2***_***HE***_, ***T3***_***HE***_) for all users without parameter tuning, whose values were from the experimental condition with the largest metabolic benefit in Ding et al. where the effect of four different sets of timing parameters were compared for an exosuit assisting hip extension [35]. Unlike the multi-articular controller, these timing parameters are represented as percentage of a gait cycle segmented by maximum hip flexion event (% GC_MHF_) detected by the thigh IMUs; note that the maximum hip flexion happens approximately 12% earlier than heel strike (i.e. % GC_MHF_ ≈ % GC - 12%) [35]. As shown in Fig. 3, the controller first detected the maximum hip flexion using the thigh IMU and used this event as the start of the controller (i.e. ***T0***_***HE***_ = 0% GC_MHF_). Then, the motor was controlled to shorten the cable up to ***PosOffset***_***HE***_ at a constant speed of 800 mm s^− 1^, which was the maximum cable speed of the hip extension actuator. After reaching ***PosOffset***_***HE***_, the controller maintained this cable position until 7% GC_MHF_ (i.e. ***T1***_***HE***_ = 7% GC_MHF_). Then, the motor further retracted the cable up to ***PosMax***_***HE***_ until 28% GC_MHF_ (i.e. ***T2***_***HE***_ = 28% GC_MHF_), and held the cable in this position until 34% GC_MHF_ (i.e. ***T3***_***HE***_ = 34% GC_MHF_). Finally, the motor released the cable to its zero position, using the maximum cable speed of 800 mm s^− 1^ similarly to the multi-articular controller. The ***PosOffset***_***HE***_ and ***PosMax***_***HE***_ were adjusted at the end of each gait cycle, to deliver a peak force of 10 N (equivalent joint moment of approximately 1 Nm) between ***T0***_***HE***_ and ***T1***_***HE***_ and a peak force of 300 N (equivalent joint moment of approximately 30 Nm) between ***T1***_***HE***_ and ***T3***_***HE***_, respectively.

### Augmentation-power-based control parameter tuning

In order to customize the multi-articular assistance for each individual, an online parameter tuning method was developed which searches the control parameters that maximize the positive augmentation power delivered at the ankle. This assumes that the positive ankle augmentation power can be an indicator of the magnitude of assistance delivered at the ankle, which in turn may have a positive correlation with the corresponding metabolic benefit [6, 10, 19, 29–33]. Of note, in this study the average positive augmentation power was calculated by dividing the positive augmentation work over a gait cycle by the stride time. The positive augmentation work may also indicate the amount of assistance delivered to the joint, but it may significantly vary with the wearer’s cadence. In contrast, positive power is less affected by variability in cadence, making it a more robust objective metric for control parameter tuning (See Additional file 1 for further discussion).

#### Tuning parameter selection

Among the control parameters defining the cable position profile of the multi-articular controller, ***T1***_***MA***_ (onset timing of the active cable retraction) and ***D***_***MA***_ (***T2***_***MA***_ - ***T1***_***MA***_; duration of the active cable retraction) were selected as the parameters to be tuned for each individual. As highlighted in green in Fig. 2, these parameters play an important role in determining the cable position profile during the active cable retraction phase, where the majority of ankle assistance is delivered during push off. In addition, in pilot experiments ***T1***_***MA***_ and ***D***_***MA***_ showed higher sensitivity to the changes in positive ankle augmentation power than other control parameters, highlighting the importance of customization of these parameters. The initial parameter ranges were set to 35–50% GC for ***T1***_***MA***_ and 7.5–22.5% GC for ***D***_***MA***_, where the actuation system could generate the desired level of peak assistive force (400 N) at the ankle joint. With this parameter range the multi-articular controller was capable of creating force profiles ranged approximately from 35 to 65% GC, which sufficiently covers the phase of positive biological ankle power while walking.

#### Positive augmentation power measurement

While walking with the exosuit active, the instantaneous ankle augmentation power was calculated from the ankle joint velocity (measured by the foot and shank IMUs) and the assistive force (measured by the multi-articular load cell), assuming a constant lever arm of 10 cm at the ankle. The positive augmentation work over a stride was calculated by integrating the positive area under the instantaneous power curve over a gait cycle, and finally the positive augmentation power was calculated by dividing the positive work by the stride time [6, 9, 30, 34, 36]. In this study, while each parameter setting was given to the wearer for 45 strides, the positive augmentation power for each condition was averaged over the last 30 strides (Note that, in pilot experiments, it took about 10 strides to reach a steady-state positive augmentation power value when a new set of parameters were applied).

#### Online parameter tuning algorithm

A simple online parameter tuning algorithm based on 2-D grid search similar to gradient descent was developed and used for this study. During the tuning process, subjects continuously walked with the exosuit on a treadmill, and the multi-articular controller applied 16 different parameter settings in series, searching the parameter values that maximize the positive augmentation power delivered at the ankle. First, the controller swept the initial four conditions, where ***T1***_***MA***_ was varied over 35, 40, 45, and 50% GC while ***D***_***MA***_ was held constant at 15% GC. These values were chosen by varying ***T1***_***MA***_ with 5% interval within its initial range (35–50% GC) while holding ***D***_***MA***_ constant at the mid-point of its initial range (7.5–22.5% GC). Among the four values of ***T1***_***MA***_, the controller selected the setting where the largest positive augmentation power was delivered to the ankle. Of note, due to the hardware limitations (specifically motor power), during this selection step the controller was designed to exclude certain parameter settings where the exosuit was limited from achieving a desired peak force of 400 N. Next, the controller applied another four conditions by varying ***D***_***MA***_ with 5% interval within its range (i.e. ***D***_***MA***_ = 7.5, 12.5, 17.5, and 22.5%) while holding ***T1***_***MA***_ constant at the previously selected value. Similarly, among the four values of ***D***_***MA***_, the controller selected the value with the largest positive ankle augmentation power. Following this alternate parameter search scheme, another set of exploration over both ***T1***_***MA***_ and ***D***_***MA***_ was repeated with a reduced interval of 2.5%. Finally, the parameter setting where the positive ankle augmentation power was maximized among the 16 conditions was chosen for each individual. Of note, the total number of conditions included in the tuning process was determined during pilot experiments, where we found that a modification of control parameters smaller than 2.5% GC did not induce a substantial change in the positive ankle augmentation power. In addition, the total 16 conditions allowed for the entire tuning process to be done in about 15 min, which is short enough to not induce significant fatigue of the wearer during the continuous walking trial.

This relatively simple parameter tuning algorithm presents several positive attributes. At this stage, the focus of the study was on testing the feasibility of the augmentation-power-based control parameter tuning approach, so a simple method aimed at proving the general concept was preferred, as opposed to applying a more sophisticated and efficient optimization technique. In addition, as this method sequentially varies either one of the two control parameters while holding the other constant, the tuning process is comprised of a series of single parameter sweeps. Compared to other multi-dimensional optimization techniques that may vary multiple parameters at the same time, this approach yields data that may provide insight on the individual parameter’s effect during the tuning process.

### Experimental protocol

**Fig. 4:**
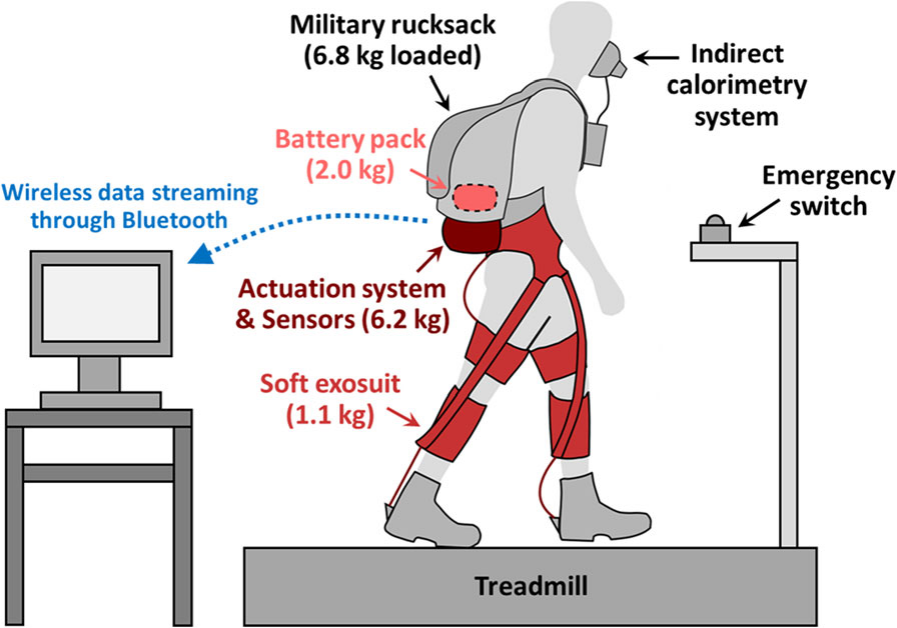
Experimental setup. The components highlighted in red, i.e. the soft exosuit, the actuation system, and the battery pack, were not included in NO-DEVICE condition

Seven healthy male adults with prior experience walking with the exosuit participated in this study (age 31.0 ± 7.3 years; mass 83.0 ± 7.9 kg; height 1.80 ± 0.04 m; mean ± SD). The Harvard Longwood Medical Area Institutional Review Board approved the study, and all participants provided written informed consent. The study consisted of a single-day experimental session, involving walking on a treadmill at 1.50 m s^− 1^ carrying a loaded military rucksack with a total mass of 6.8 kg, with and without wearing the exosuit (Fig. 4). Note that a fixed walking speed was used in this study, because variation in walking speed by itself may change the positive ankle augmentation power. The amount of load carried during this experiment was selected to not induce fatigue in the participants. At the beginning of the session, a 4-min standing trial was performed to collect steady-state standing metabolic cost. Participants then underwent a 15-min control parameter tuning process explained above. After the tuning process, participants completed three 5-min experimental conditions: loaded walking with the exosuit unpowered (EXO-OFF), loaded walking with the exosuit active using individualized parameters found by the augmentation-power-based parameter tuning (EXO-ON), and loaded walking without wearing the exosuit (NO-DEVICE). The two exosuit conditions were randomized, but NO-DEVICE condition was always completed last to minimize exosuit donning and doffing time during the session.

### Measurement and data processing

A portable indirect calorimetry system (K4b2, COSMED, Rome, Italy) was used to measure the metabolic cost of walking. Metabolic power was calculated using a modified Brockway equation [37] and averaged over the last 2 min of each condition. For each walking condition, net metabolic rate was calculated by subtracting the metabolic power during standing and then normalized to each participant’s body mass. Percent *net* metabolic benefit was calculated as the reduction in net metabolic rate for EXO-ON condition compared to NO-DEVICE condition, while *gross* metabolic benefit was calculated compared to EXO-OFF condition:

$$ Net\  Metabolic\ Benefit\ \left[\%\right]=\frac{\left(N{O}_{DEVICE}\right)-\left( EX{O}_{ON}\right)}{\left(N{O}_{DEVICE}\right)-(STANDING)}\times 100\% $$

$$ Gross\ Metabolic\ Benefit\ \left[\%\right]=\frac{\left( EXO\_ OFF\right)-\left( EXO\_ ON\right)}{\left( EXO\_ OFF\right)-(STANDING)}\times 100\% $$

Inter-subject mean and standard error of the mean (SEM) were calculated for the net metabolic rate and the percent metabolic reduction. Two-sided paired t-tests (significance level α = 0.01; MATLAB, MathWorks Inc., Natick, MA, USA) were used to test statistical significance of the difference in net metabolic rate between two conditions.

## Results

### Positive ankle augmentation power map

**Fig. 5:**
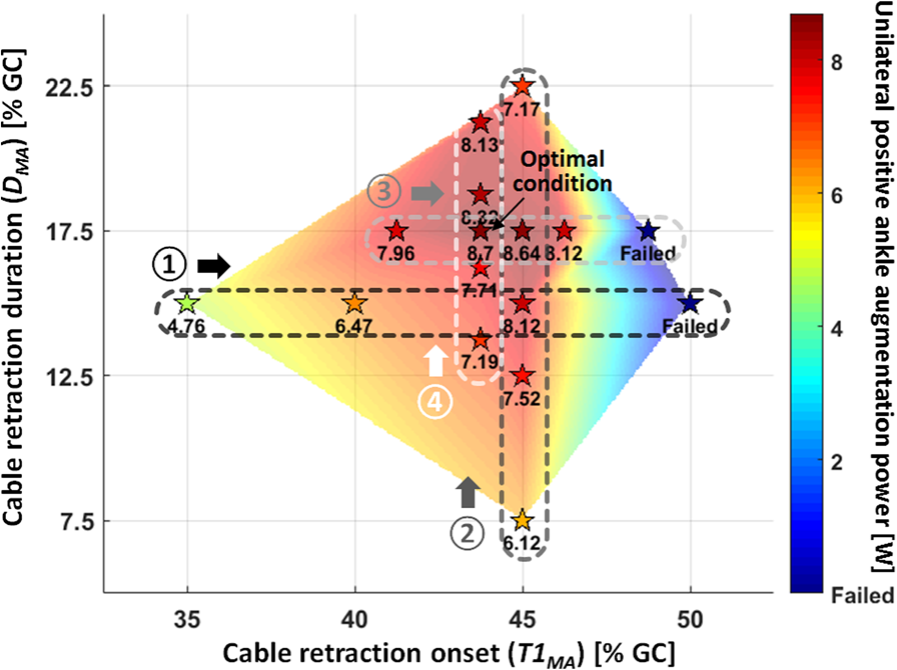
Positive ankle augmentation power map of a representative subject (S5) from the control parameter tuning process. Stars indicate the conditions that the controller explored, and the number below each star indicates the unilateral positive augmentation power delivered at the ankle for each condition in watts. The arrows with numbers in circles (①, ②, ③, and ④) indicate the sequence of the exploration, along the grouped conditions indicated by the dotted lines. The stars labelled as “Failed” indicate the conditions that were excluded as the exosuit was limited from achieving a desired peak force of 400 N

Figure 5 shows a positive ankle augmentation power map of a representative subject (S5) from the parameter tuning process. As shown, the controller explored a wide range of conditions and found the parameter set (***T1***_***MA***_ = 43.75%, ***D***_***MA***_ = 17.5%) that delivered the most positive augmentation power at the ankle (unilateral 8.70 W), which was 83% higher than the lowest condition (unilateral 4.76 W at ***T1***_***MA***_ = 35%, ***D***_***MA***_ = 15%).

### Subject-specific control parameters

**Fig. 6:**
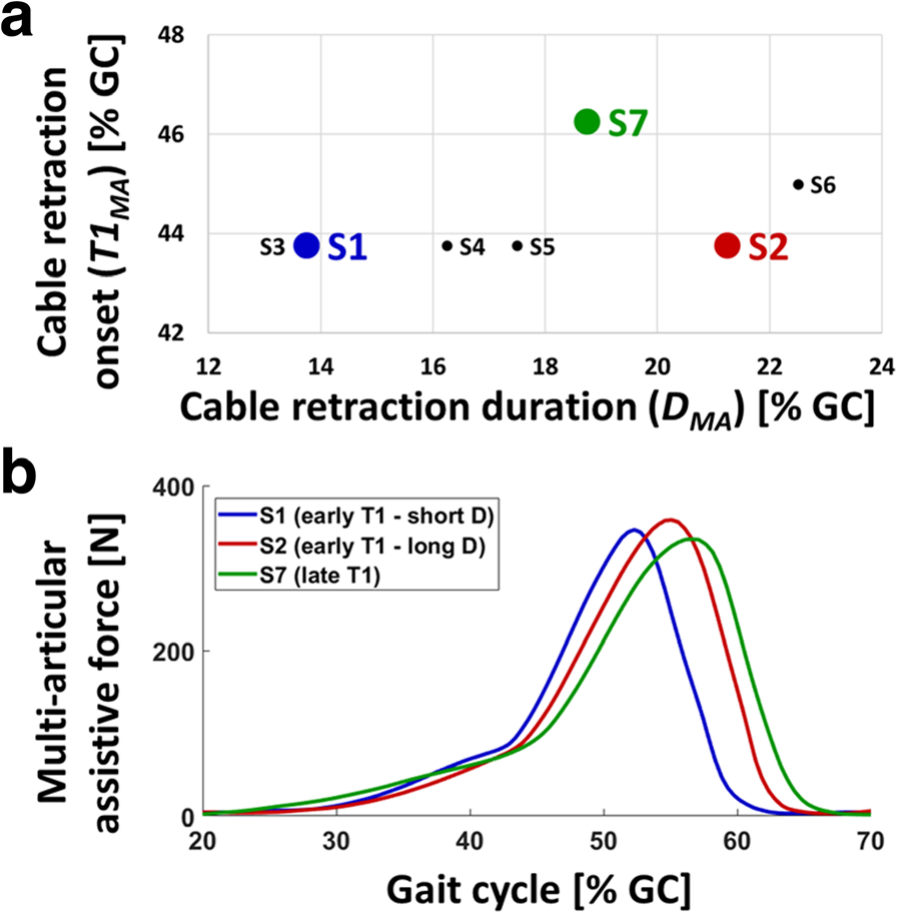
Subject-specific multi-articular assistance found by the parameter tuning method. **a** Distribution of the subject-specific parameters (T1_MA_ and D_MA_) across the seven participants (S1 to S7). **b** Resultant multi-articular assistive force profiles of three representative subjects: S1 (blue), S2 (red), and S7 (green)

Figure 6 shows the subject-specific control parameters found by the parameter tuning method and the resultant force profiles. As shown in Fig. 6a, the wearer-specific cable retraction onset (***T1***_***MA***_) ranged from 43.75 to 46.25% of a gait cycle, whereas the cable retraction duration (***D***_***MA***_) ranged from 13.75 to 22.5% of a gait cycle across the participants. These differences in cable retraction timing resulted in a wide range of subject-specific force profiles for multi-articular assistance, as shown in Fig. 6b.

### Metabolic cost

**Fig. 7:**
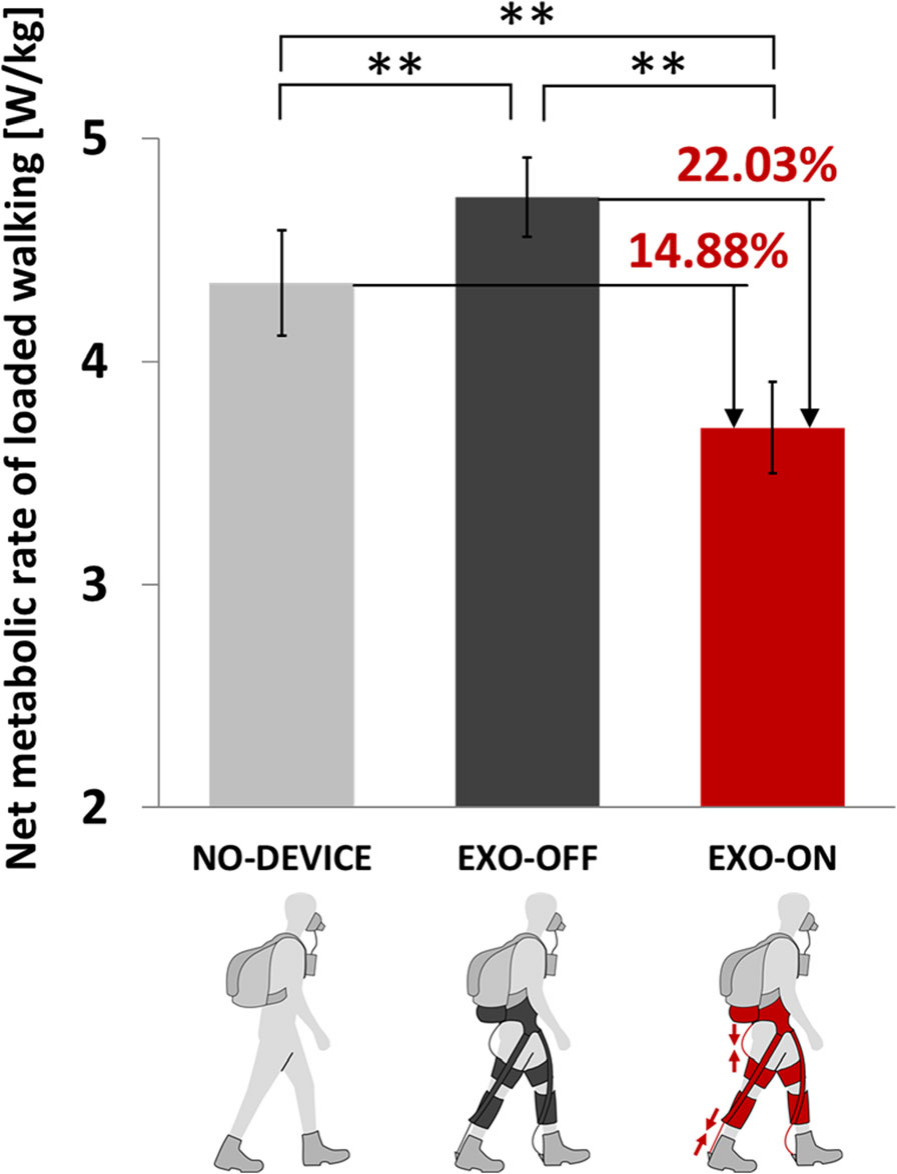
Metabolic cost of load carriage for the three experimental conditions. Solid bars indicate inter-subject mean of net metabolic rate of loaded walking, while error bars indicate SEM. Double asterisks (**) indicate that the difference between the two conditions is statistically significant (paired t-test; *n* = 7; *P* < 0.01). For EXO-ON, the net metabolic rate of loaded walking was significantly reduced by 14.88 ± 1.09% compared to NO-DEVICE (*P* = 5 × 10–5) and by 22.03 ± 2.23% compared to EXO-OFF (*P* = 2 × 10–5)

Walking with the autonomous multi-joint soft exosuit with individualized parameters significantly improved the energy economy of load carriage for all participants (Table 1). As shown in Fig. 7, the net metabolic rate of loaded walking in NO-DEVICE, EXO-OFF, and EXO-ON conditions were 4.35 ± 0.24 W/kg, 4.74 ± 0.18 W/kg, and 3.70 ± 0.21 W/kg (mean ± SEM), respectively. This corresponds to a significant *net* metabolic reduction of 14.88 ± 1.09% (paired t-test, *P* = 5 × 10^− 5^) and a significant *gross* metabolic reduction of 22.03 ± 2.23% (*P* = 2 × 10^− 5^).

**Table 1:** Metabolic result for each participant

Subject	STANDING [W/kg]	NO-DEVICE [W/kg]	EXO-OFF [W/kg]	EXO-ON [W/kg]	Net metabolic benefit [%]	Gross metabolic benefit [%]
S1	1.267	5.039	5.643	4.423	16.34%	27.88%
S2	1.025	4.975	5.172	4.457	13.12%	17.26%
S3	1.331	6.306	6.537	5.334	19.55%	23.11%
S4	1.707	6.258	6.613	5.705	12.15%	18.50%
S5	1.319	6.288	6.681	5.728	11.27%	17.77%
S6	1.486	4.929	5.768	4.394	15.53%	32.08%
S7	1.572	6.362	6.444	5.586	16.18%	17.60%
Mean (± SEM)					14.88% (± 1.09%)	22.03% (± 2.23%)

## Conclusion

In this paper, we present an autonomous multi-joint soft exosuit and an online parameter tuning approach that customizes assistance for each individual based on the positive power delivered at the ankle. The parameter tuning method was capable of automatically finding the wearer-specific control parameters using exosuit sensors, demonstrating its potential to customize an assistive device outside of the lab so as to maximize positive augmentation power at the ankle. The subject-specific control parameters resulted in a wide range of assistance profiles, which supports the growing recognition of the importance of such control individualization for assistive devices. Additionally, the autonomous multi-joint exosuit with the subject-specific control parameters significantly reduced the net metabolic cost of loaded walking by 14.88%, relative to walking without wearing the device.

Despite a significant metabolic benefit, there are a number of limitations of this study worth mentioning. First, although the capability of the control parameter tuning method to maximize the positive ankle augmentation power was demonstrated, the amount of metabolic improvement produced by using the subject-specific parameters was not clear. At this stage, the focus was on proving the general feasibility of the approach, and the experiment that we conducted was not able to isolate the effect of the control parameter tuning. Follow-up studies will investigate the metabolic landscape versus control parameters, bridging the gap between the augmentation-power-based parameter tuning at the ankle and the whole-body energetics. Second, the parameter tuning algorithm was based on a simple grid search similar to gradient descent, which may be vulnerable to existence of local minima or measurement noise. Future research will explore the use of statistical optimization algorithms which can search the global optimum in a large parameter space, such as Bayesian optimization [20] or simulated annealing [38], to make the tuning process more reliable and robust. In addition, the joint and muscle-tendon level mechanisms that contributed to this high metabolic reduction is not yet clear due to limited biomechanical and physiological measurements. In follow-up studies, we will include more comprehensive measurements, such as 3-D motion capture, electromyography, and ultrasound imaging, to further investigate how the exosuit and the wearer interact with each other. Lastly, whereas only a specific walking condition was tested in this study, future studies will evaluate the efficacy of the device in various conditions, such as walking at different walking speeds with different loads. This may provide insights on developing a parameter tuning method suitable for overground walking where the wearer may continuously change their walking speed, enabling potential use of this parameter tuning method for patient populations [39, 40].

## Additional file

Additional file 1: Variability of positive augmentation power and work with cadence. (PDF 285 kb)

## Abbreviations

% GC: Percentage of a gait cycle (segmented by heel strike)
% GC_MHF_: Percentage of a gait cycle segmented by maximum hip flexion
HE (subscription): Parameters for hip extension controller
IMU: Inertial measurement unit
MA (subscription): Parameters for multi-articular controller
SD: Standard deviation
SEM: Standard error of the mean
