# Supplementary material for: Autonomous multi-joint soft exosuit with augmentation-power-based control parameter tuning reduces energy cost of loaded walking
Source: J Neuroeng Rehabil. 2018 Jul 13;15:66. doi: 10.1186/s12984-018-0410-y (PMC6044002; doi:10.1186/s12984-018-0410-y)
Supplement: Supplementary file 1 — Variability of positive augmentation power and work with cadence. (PDF 285 kb) [file 12984_2018_410_MOESM1_ESM.pdf]

Supplementary Materials for

Autonomous multi-joint soft exosuit with augmentation-power-based control parameter tuning reduces energy cost of loaded walking

Sangjun Lee,[1,2] Jinsoo Kim,[1,2*] Lauren Baker,[1,2*] Andrew Long,[1,2]
Nikos Karavas,[1,2] Nicolas Menard,[1,2] Ignacio Galiana,[1,2] Conor J. Walsh[1,2†]

[1]John A. Paulson School of Engineering and Applied Sciences, Harvard University, Cambridge, MA, USA
[2]Wyss Institute for Biologically Inspired Engineering, Harvard University, Cambridge, MA, USA

*These authors contributed equally to this work.

†Corresponding author:

Dr. Conor J. Walsh

John L. Loeb Associate Professor of Engineering and Applied Sciences at the John A. Paulson School of Engineering and Applied Sciences, Harvard University, Cambridge, MA, USA

Core Faculty Member at the Wyss Institute for Biologically Inspired Engineering, Harvard University, Cambridge, MA, USA

walsh@seas.harvard.edu

The PDF file includes:

- Supplementary text
- Table S1. Changes in positive power and positive work with respect to changes in cadence.
- Fig. S2. Changes in cadence of each participant during the control parameter tuning process.
- References (41)

Variability of positive augmentation power and work with cadence

In this study, the positive ankle augmentation power was used as the objective metric of the online control parameter tuning method. One may argue that the positive augmentation work (i.e. without dividing by the stride time) may also be an indicator of the magnitude of assistance delivered at the joint; however, given that the assistance profile was defined based on percentage of a gait cycle, the positive work may vary significantly with the wearer's cadence. In contrast, the positive augmentation power is less affected by variability in cadence, making it a more robust objective metric.

To be specific, Umberger and Martin (2007) [41] reported that the positive ankle power profile varied when cadence changed, which resulted -6.3% to +15.6% changes in average positive ankle power (Row (a) in Table S1). Meanwhile, the changes in positive work would be -21.6% to +45.2% (Row (c) in Table S1) which was much more variable than the positive power, as the changes in stride time (Row (b) in Table S1) are multiplied to the changes in positive power.

	Different cadence conditions	-20%	-10%	Preferred	+10%	+20%
(a)	Average ankle positive *POWER* over a stride [W kg^{-1}]<br>(Adopted from Table 2 in Umberger 2007)	0.37<br>(+15.6%)	0.34<br>(+6.3%)	0.32	0.29<br>(-9.4%)	0.30<br>(-6.3%)
(b)	Average stride time [sec]<br>(Adopted from Table 1 in Umberger 2007)	1.38	1.23	1.10	1.00	0.92
(c)	Average ankle positive *WORK* over a stride [J kg^{-1}]<br>(Calculated from (a) and (b))	0.511<br>(+45.17%)	0.418<br>(+18.75%)	0.352	0.290<br>(-17.61%)	0.276<br>(-21.59%)

Table S1. Changes in positive power and work with respect to changes in cadence, using actual data in [41].

Of note, in fact the average cadence of each participant during the parameter tuning process did not substantially vary in this study, so we expect neither the positive power nor the positive work was significantly affected by the cadence. To be specific, as shown in Fig. S2, the changes in average cadence across different conditions was less than ±3% for all participants. By applying a quadratic polynomial regression to predict the changes in positive ankle power, which is the same method with [41], the estimated changes in positive power induced by this amount of cadence change would be only -1.5% to +1.1%.

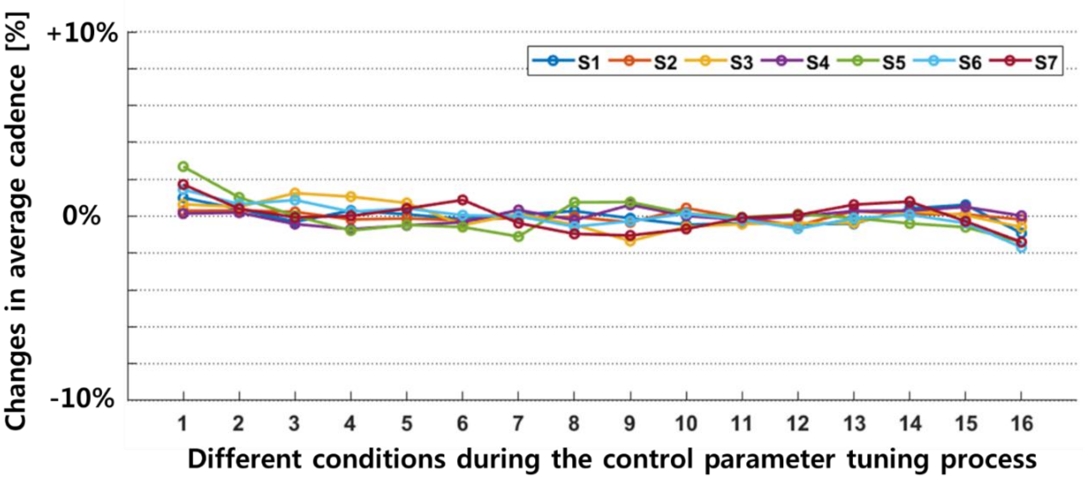

Fig. S2. Changes in cadence of each participant over the 16 conditions during the control parameter tuning process. Each colored line indicates each subject. Each value was calculated as a percentile change with respect to each participant's average cadence.

References

[41] B. R. Umberger and P. E. Martin, "Mechanical Power and Efficiency of Level Walking with Different Stride Rates," *J Exp Biol*, vol. 210, 2007.
